# Advanced light-trapping effect of thin-film solar cell with dual photonic crystals

**DOI:** 10.1186/s11671-015-0912-5

**Published:** 2015-05-09

**Authors:** Anjun Zhang, Zhongyi Guo, Yifei Tao, Wei Wang, Xiaoqin Mao, Guanghua Fan, Keya Zhou, Shiliang Qu

**Affiliations:** School of Computer and Information, Hefei University of Technology, 193# Tunxi Road, Hefei, 230009 Anhui Province China; Department of Optoelectronics Science, Harbin Institute of Technology, Weihai Campus, 2# Wenhua West Road, Weihai, 264209 Shandong Province China; Department of Physics, Harbin Institute of Technology, 91# West Dazhi Street, Harbin, 150001 Heilongjiang Province China

**Keywords:** Thin film solar cells, Dual photonic crystals, Optimized shape, Light trapping

## Abstract

A thin-film solar cell with dual photonic crystals has been proposed, which shows an advanced light-trapping effect and superior performance in ultimate conversion efficiency (UCE). The shapes of nanocones have been optimized and discussed in detail by self-definition. The optimized shape of nanocone arrays (NCs) is a parabolic shape with a nearly linearly graded refractive index (GRI) profile from the air to Si, and the corresponding UCE is 30.3% for the NCs with a period of 300 nm and a thickness of only 2 μm. The top NCs and bottom NCs of the thin film have been simulated respectively to investigate their optimized shapes, and their separate contributions to the light harvest have also been discussed fully. The height of the top NCs and bottom NCs will also influence the performances of the thin-film solar cell greatly, and the result indicates that the unconformal NCs have better light-trapping ability with an optimal UCE of 32.3% than the conformal NCs with an optimal UCE of 30.3%.

## Background

Due to the abundance and a nonpoisonous feature, Si-based solar cells are still dominating the photovoltaic market. As silicon is a kind of an indirect band gap material, the light absorption of Si is poor. In order to meet the requirement of light harvesting, the thickness of Si active layer should be no less than 100 μm. What is more, costly processes, such as purification and crystallization, must be implemented to ensure the efficient collection of photon-generated carriers. As a result, the cost of bulk of Si solar cells is high, which hinders the commercialization of Si solar cells. To overcome this problem, thin-film solar cells have been proposed, which not only reduces the cost of Si solar cells but also improves the collection of carriers because of the reduced diffusion length [1]. However, the reduction of the thickness of Si solar cells leads to poor absorption of the longer wavelengths (near the band gap) [2]. The absorption length of ζ grows rapidly as the wavelength increases [3], which exceeds 2 μm at a wavelength of 700 nm and reaches 5 μm at a wavelength of 800 nm. The absorption length of light in longer wavelengths exceeds the thickness of the thin-film Si, which leads to the poor absorption. In order to solve this issue, recently, lots of light-trapping structures, including randomly structured surfaces [4,5], periodic gratings [6-8], photonic crystals [9], plasmonic structures [10-13], Si nanowire arrays (SiNWs) [14-16], and Si nanocone arrays(SiNCs) [17,18] have been proposed and investigated widely. The SiNWs layer can reduce the reflection efficiently because it can serve as a buffer layer to diminish the refractive index mismatch between the air and the Si wafer [16]. The SiNCs can further reduce the reflection because the refractive index changes gradually from the top to the bottom of the SiNCs [17]. Similarly, nanodome arrays [19] have also been investigated by using the same strategy to reduce the reflection. Kuo and Hsieh [20] have proposed a novel way to optimize the shape of ZnO nanocone arrays (NCs) located at the front surface of CZTS solar cells. By changing the parameter of ‘order of taper (OT),’ the shape of the NCs could be changed for optimizing the performance of the solar cell. The dual photonic crystal-based solar cells can trap the incident light by diffraction, the Bloch modes coupling, the excitation of SPRs, and multiple scattering effects [21-23]. Thus, the dual photonic crystal cells have great potentials in the field of light harvesting. However, the influences of the shapes and the heights of the top NCs and bottom NCs on the light-trapping performance have not been investigated and discussed systematically.

In this paper, we have proposed a thin-film solar cell with dual photonic crystals. We have systematically optimized the shape of the top NCs and bottom NCs by self-definition. For conformal NCs, the parabolic shape with linearly graded refractive index (GRI) profile is the best choice. The optimized shape of top NCs is also parabolic while that of bottom NCs is needle-shaped. The heights of the NCs have also been optimized. The optimal height of conformal nanostructures is 300 nm which balances the reflection loss and parasitic loss, and the resulted ultimate conversion efficiency (UCE) is 30.3% nearly. For top NCs, the optimized height is also 300 nm because of the optimized light-trapping effects. When the height of the top NCs is set as 300 nm, the optimized height of the bottom NCs can be obtained as 100 nm, in which the corresponding UCE is 32.3% that is higher than the performance of the traditional conformal NCs (the heights of both the top and bottom NCs are the same as 300 nm)-based solar cells.

## Methods

**Figure 1:**
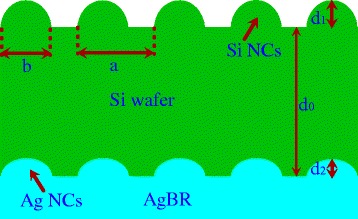
The schematic of the periodical nanostructures of the dual photonic crystal-based thin-film solar cell. The corresponding parameters are denoted in the figure. NCs, nanocone arrays.

**Figure 2:**
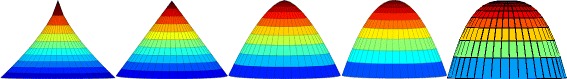
The shapes of the NCs with different OTs of 0.7, 1, 1.6, 2, and 3, respectively.

Figure 1 shows the schematic of the simulated dual photonic crystal-based solar cell architecture in 2D cross-section. The thickness of the Si wafer d_0_ is set to be 2,000 nm. The d_1_ and d_2_ denote the height of the Si NCs (top NCs) and Ag NCs (bottom NCs), respectively, and each of them can be changed individually. The variable ‘a’ denotes the period of the square lattice and the ‘b’ represents the value of the bottom diameters of the NCs. The periods (‘a’) and the bottom diameters of the NCs for the top NCs (‘b’) and those of the bottom NCs are the same. Thus, the filling ratio (FR) can be defined as: FR = πb^2^/4a^2^. Here, we defined a parameter, OT, and the shapes of various-shaped nanostructures can be defined by OT:

1 $$ r=R\sqrt[OT]{\left(H-z\right)/H},\cdot \mathrm{and}\;{x}^2+{y}^2={r}^2\left(0\le z\le H\right) $$

where *r* is the radius of the nanostructure in the XY-plane at the height of *H*, and *R* is the bottom radius of the NCs. Therefore, according to Equation 1, we can obtain the shapes of the NCs with different OTs. Figure 2 illustrates the schematic of NCs with different OTs of 0.7, 1.0, 1.6, 2, and 3, respectively.

We have performed the 3D numerical simulation by finite-difference time-domain (FDTD) method. The incident light is set to be x-polarized and propagating along the z-axis direction. The wavelength (λ) of incident lights lies in the ranges of 300-1,127 nm corresponding to the band gap of the Si (1.1 eV). The real and imaginary part of the refractive index of Si and Ag are obtained from ref [24]. In order to evaluate the light-harvesting ability of the designed solar cells, some parameters are defined. The absorption in the Si or Ag can be defined by the following equation [25]:

2 $$ {A}_{\mathrm{Si},\mathrm{A}\mathrm{g}}=\frac{{\displaystyle \iiint {Q}_{\mathrm{Si},\mathrm{A}\mathrm{g}}\left(\mathrm{x},\mathrm{y},\mathrm{z},\uplambda \right)dV}}{E_{\mathrm{t}}} $$

where *Q*_Si,Ag_(x, y, z, λ) refers to the absorbed energy at a specified point when the wavelength of the incident light equals λ and *E*_t_ denotes the total energy of the incident light. The *Q*_Si,Ag_(x, y, z, λ) can be calculated by the following equation [26]:

3 $$ {Q}_{\mathrm{Si},\mathrm{A}\mathrm{g}}\left(\mathrm{x},\mathrm{y},\mathrm{z},\uplambda \right)=\frac{1}{2}\omega \mathrm{I}\mathrm{m}\left(\upvarepsilon \left(\uplambda \right)\right)\left|E\left(\mathrm{x},\mathrm{y},\mathrm{z},\uplambda \right)\right|{}^2 $$

where *E*(x, y, z, λ) refers to the electric field intensity at point (x, y, z) when the wavelength of the incident light equals λ. Im(ε(λ)) represents the imaginary part of the wavelength-depended relative dielectric constant. The variable *ω* denotes the angular frequency of the incident light.

In order to evaluate the overall performance of the solar cell in the whole wavelength region, we defined the UCE of *η* which can be expressed by the following equation [25]:

4 $$ \eta =\frac{{\displaystyle {\int}_{310\mathrm{nm}}^{\uplambda \mathrm{g}}}I\left(\uplambda \right){\mathrm{A}}_{\mathrm{Si}}\frac{\uplambda}{\uplambda_{\mathrm{g}}}\mathrm{d}\uplambda}{{\displaystyle {\int}_{310\mathrm{nm}}^{4,000\mathrm{nm}}}I\left(\uplambda \right)\mathrm{d}\uplambda} $$

where *λ*_g_ is 1,127 nm which corresponds to the band gap energy of Si (1.1 eV), and *I*(λ) refers to the spectral irradiance at AM 1.5 direct normal conditions in W/m^2^/nm. We also assume that one photon whose energy is greater than the bad gap energy can only generate one electron-hole pair while the excessive energy will be lost in the form of heat.

**Figure 3:**
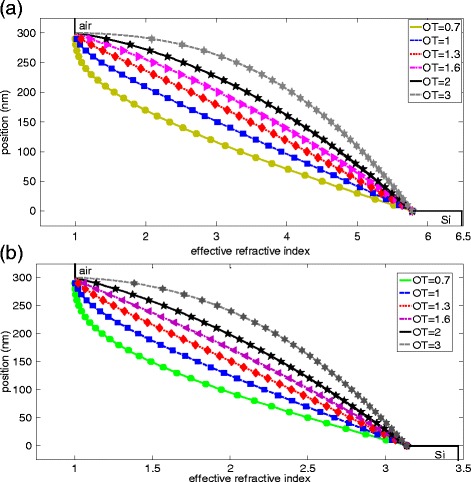
The GRI profiles of the sample at the wavelengths of **(a)** 300 nm and **(b)** 800 nm. OT, order of taper.

The averaged reflection loss and parasitic absorption loss are proposed in order to investigate the optical loss mechanism in our structure. These two parameters can be calculated by the following equations:

5 $$ {R}_{\mathrm{av}}=\frac{{\displaystyle {\int}_{310\mathrm{nm}}^{1,200\mathrm{nm}}}\mathrm{I}\left(\uplambda \right)R\left(\uplambda \right)\mathrm{d}\uplambda}{{\displaystyle {\int}_{310\mathrm{nm}}^{1,200\mathrm{nm}}}\mathrm{I}\left(\uplambda \right)\mathrm{d}\uplambda} $$

6 $$ {\mathrm{PL}}_{\mathrm{av}}=\frac{{\displaystyle {\int}_{310\mathrm{nm}}^{1200\mathrm{nm}}}\mathrm{I}\left(\uplambda \right){\mathrm{A}}_{\mathrm{A}\mathrm{g}}\left(\uplambda \right)\mathrm{d}\uplambda}{{\displaystyle {\int}_{310\mathrm{nm}}^{1200\mathrm{nm}}}\mathrm{I}\left(\uplambda \right)\mathrm{d}\uplambda} $$

where *R*(λ) refers to the wavelength-dependent reflectance and *A*_Ag_(λ) denotes the absorption in Ag. These two parameters are defined in order to evaluate the overall loss in the whole wavelength region. As the GRI effect plays a very important role in antireflection in our nanostructure, the effective refractive index at a specific position should be calculated by the following equation [20]:

7 $$ {n}_{\mathrm{eff}}={\left[{n}_{\mathrm{Si}}^2\times \mathrm{F}\mathrm{R}+{n}_{\mathrm{air}}^2\times \left(1-\mathrm{F}\mathrm{R}\right)\right]}^{\frac{1}{2}} $$

where *n*_Si_ and *n*_air_ are the real parts of the refractive index of Si and air. FR refers to the filling ratio at a specific position which can be defined as: FR = πr^2^/4a^2^. Therefore, we can obtain the GRI profiles of the NCs sample with different OTs from the air to the Si layer. Figure 3 illustrates the GRI profiles of the sample at short wavelength region (300 nm) and longer wavelength region (800 nm).

## Results and discussion

**Figure 4:**
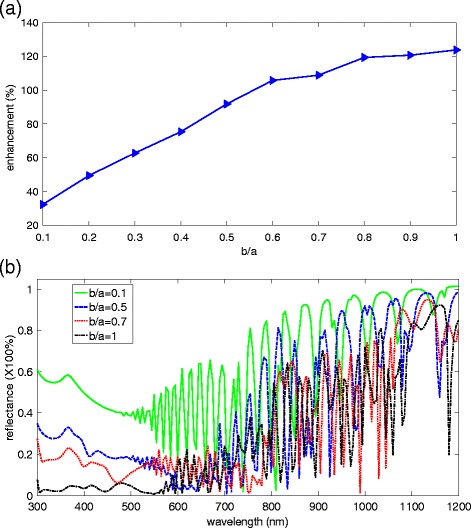
The optimization of the bottom diameter of NCs. **(a)** The relationship between b/a and the relative enhancement factor. **(b)** The reflectance spectra of NCs with different values of b/a.

The geometry parameters of NCs strongly influence the optical harvesting ability of these Si structures [27,28]. Thus, the structure parameters should be optimized and discussed. The bottom diameter of NCs is an important factor influencing the light harvest [29]; as a result, the bottom diameter of b should be discussed firstly. The period and height of NCs are set to be 300 nm [20] to ensure efficient light trapping, and the OT is set as 2. Figure 4a shows the enhancement factor of UCE as a function of b/a. The enhancement factor is defined as (η_NCs_ − η_Si_)/η_Si_, where η_NCs_ expresses the UCE of the designed NC solar cell and η_Si_ expresses the UCE of the planar Si solar cell with the thickness of 2,300 nm (the same value as that of NC solar cell), respectively. We can observe that the enhancement factor reaches its maximum when b = a. The corresponding UCE and enhancement factor are 30.2% and 123.8%, respectively. Figure 4b depicts the reflectance spectra of NCs with different b/a. We can observe that the reflectance in the short wavelength region is bigger than 50% when b/a = 0.1. The reflectance in the short wavelength region (300 ~ 600 nm) decrease as the b/a (from 0.1 ~ 1.0) increase, and it reaches to the minimum when b/a = 1.0, which can be attributed to the GRI effect [17].

**Figure 5:**
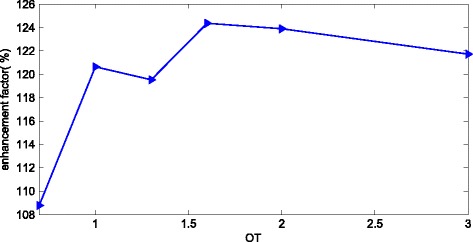
The relationship between the enhancement factors of the UCE and OT of conformal NC solar cell.

We keep a = b = 300 nm, and the d_1_ and d_2_ are also set to be 300 nm. By changing the parameter ‘OT’ from 0.7 to 3, we can obtain the UCEs of the NC solar cell as shown in Figure 5. We can observe that the optimal value for OT is 1.6. In such a situation, the corresponding enhancement factor is about 124% and the corresponding UCE is 30.3%.

Our designed dual photonic crystals can enhance the UCE drastically with the enhancement factors of 124% comparing with the performance of the planar Si solar cell with the same depth. There are several light-trapping mechanisms in the dual photonic crystal. Besides that of the GRI effect [17] of the top NCs which can suppress the surface reflection effectively, there are other kinds of light-trapping strategies that should be mentioned here, such as the localized surface plasmon resonance (LSPR) of the bottom Ag NCs [25] and the Bloch modes coupling in the Si conformal NCs [30], which can also trap the incident lights in our designed dual photonic crystal thin-film solar cell. The generated LSPR in the surface of the bottom Ag NCs can be scattered out of the Ag NCs again to the Si layer with the nonzero lateral wave vector, which will enlarge the light path in the Si layer, so the absorption of the active materials (Si) will be enhanced. According to the photonic crystal theory, there are some Bloch modes in the Si conformal NCs and the diffracted light can couple into these modes and propagate with nonzero lateral wave vector, which will also enlarge the light path in the Si layer, so as to enhance the UCE of the NC thin-film solar cell.

**Figure 6:**
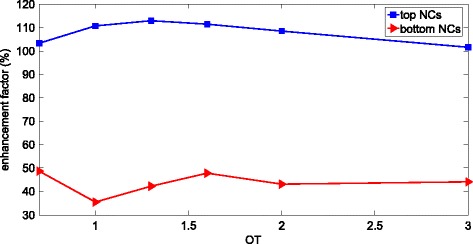
The enhancement factor of top NCs and bottom NCs as functions of OT. NCs, nanocone arrays; OT, order of taper.

In order to investigate the contribution of the top NCs and bottom NCs and optimal OT for the top NCs and bottom NCs, we have also simulated the Si solar cells with only top NCs and bottom NCs, respectively. For Si thin cells with only top NCs, d_1_ = 300 nm and there is no Ag back reflector at the backside of the sample so that we can investigate the antireflection property of top NCs merely. For Si thin-film solar cells with only bottom NCs, d_2_ is also set to be 300 nm. For both of the top NCs and bottom NCs samples, a = b = 300 nm. Figure 6 illustrates the enhancement factors (comparing with the planar silicon thin-film solar cell as mentioned before) of top NCs sample and bottom NCs sample, respectively, and it can be easily observed that the enhancement factor of top NCs is much higher than that of bottom NCs. This result indicates that the antireflection of top NCs plays a more important role in light trapping for the 2,000 nm Si wafer. This is mainly due to the high refractive index of Si, which leads to high reflection at the Si/air interface.

**Figure 7:**
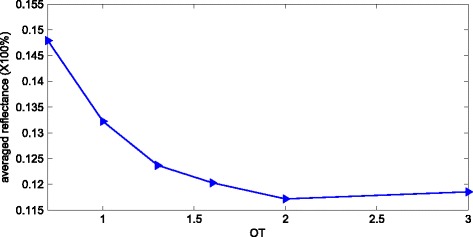
The relationship between averaged reflection and OT. OT, order of taper.

For the sample only with top NCs, the optimal value for OT is about 1.3 ~ 1.6 as shown in Figure 6, and the corresponding enhancement factor and UCE are 113% and 28.74%, respectively. The averaged reflectances of the samples with different OTs obtained by Equation 5 are shown in Figure 7. The averaged reflection keeps decreasing until OT reaches 2 and then increases. And the minimum averaged reflection is 11.7% when OT = 2. It can be seen clearly that the top NCs can reduce the reflection dramatically, which can be attributed as the GRI effect, multiple scattering, and waveguide modes coupling [19]. In the longer wavelength region (500-1200 nm), these three kinds of mechanisms are applicable. However, in the shorter wavelength region (300-500 nm), the GRI effect will be degenerated because the incident wavelength is comparable with the nanostructure period (a = 300 nm), so the main light-trapping mechanisms are the multiple scattering and waveguide modes coupling.

Generally speaking, owing to the GRI effect, if the GRI profile is more linear, the reflection caused by refractive index mismatch will become lower. According to Figure 3, the GRI profile is most linear when OT = 1.6, which leads to the least reflection caused by refractive index mismatch. However, as shown in Figure 7, the averaged reflectance reaches to minimum when OT = 2, which seems contradictory with the existing GRI effect. The reason leading to this phenomenon is that the dominated antireflection mechanisms in the shorter wavelength region (300-500 nm) are waveguide modes coupling and multiple scattering. With the increases of OT, the top diameters of the NCs will be enlarged as depicted in Figure 2, which will enhance the guide modes coupling [25,29] in the top NCs. Therefore, the averaged reflectance of the NCs with OT = 2 is lower than that of NCs with OT = 1.6. However, when OT changes from 2 to 3, the guide modes coupling will be further enhanced in theory, but the GRI profile deviates greatly from linear status. Consequently, the waveguide modes coupling cannot compensate the reflection caused by refractive index mismatch; thus, the averaged reflectance increases to a little larger value as shown in Figure 7.

**Figure 8:**
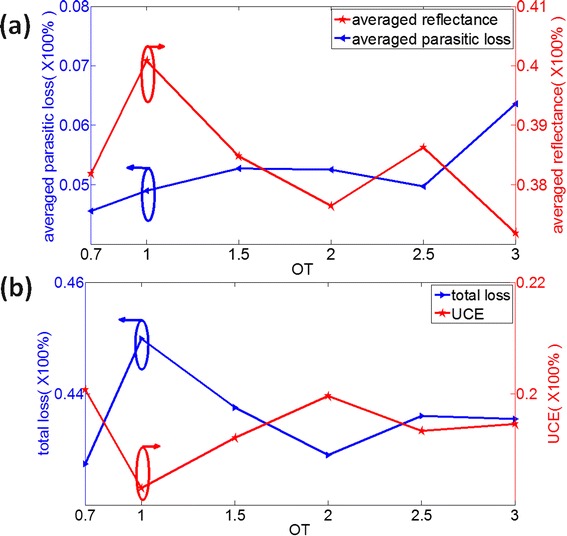
The optimization of the shape of bottom NCs. **(a)** The parasitic loss of Ag NC structure and the averaged reflectance and **(b)** the total loss and UCE of samples only with bottom NCs as a function of OT. OT, order of taper; UCE, ultimate conversion efficiency.

For the sample only with the bottom Ag NCs structure, the optimal OT for bottom NCs is 0.7 as shown in Figure 6, and the corresponding UCE and enhancement factor are 20.07% and 48.6%, respectively. We have investigated the averaged reflectance and averaged parasitic loss (Equation 6) to get a clear insight of the physical mechanism. As the front surface is planar, so the averaged reflectance is higher than 38% nearly, but it can characterize the light-trapping ability which is caused by the excitation of SPR. However, the excitation of SPR can lead to higher parasitic loss, which is harmful to the light harvesting. Figure 8a illustrates the average reflectance and averaged parasitic loss of Ag NCs structure, respectively. Figure 8b shows the total loss and the UCE, where we can find that the bottom NCs with lower total loss will have higher UCE. The bottom NCs with OT = 0.7 illustrates the lowest total loss, which leads to the best optical performance. According to Figure 8a, the averaged parasitic loss of bottom NCs with OT = 0.7 is the lowest and it also has a moderate averaged reflectance, which leads to the lowest total loss. This result indicates that the bottom NCs with OT = 0.7 can efficiently trap the incident light with the lowest parasitic loss; as a result, it has the optimal UCE of 20.07%.

For the conformal-structure thin film with dual photonic crystal of top NCs (with OT = 1.6, d_1_ = 300 nm) and bottom NCs (with OT = 1.6, d_2_ = 300 nm), both of the NCs have good optical absorption characteristics, which indicates the NCs with OT = 1.6 can act as not only top gratings but also bottom gratings of the thin-film solar cell. In conclusion, the nanostructure in conformal NCs should be chosen as parabolic shape with nearly linearly GRI profile, and the performance is shown in Figure 5.

The dual grating nanostructures with OT = 2 for top NCs and OT = 0.7 for bottom NCs is also investigated in simulation, and the corresponding UCE is 30% nearly. When OT = 2, the top NCs shows the best antireflection property and OT = 0.7 is the optimal shape for the bottom NCs as discussed above. The resulted UCE surpasses that of conformal NCs with OT = 0.7 (28.6%), but it is a little less than those of conformal NCs with OT = 2 (30.2%) and OT = 1.6 (30.3%). This result indicates that although we can optimize the shape of top NCs and bottom NCs for further improving the light-trapping ability and the UCE of the thin film, the integration of top NCs and bottom NCs with corresponding optimal parameters cannot reach the best results because top NCs and bottom NCs may influence each other.

**Figure 9:**
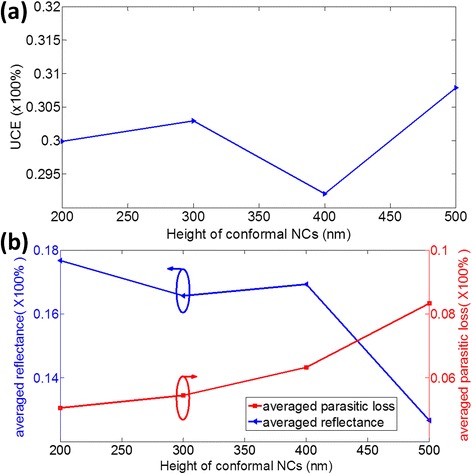
The optimization of the height of conformal NCs. **(a)** The relationship between H of NCs and UCEs. **(b)** The averaged reflectance and averaged parasitic loss of NCs with different heights. NCs, nanocone arrays; UCE, ultimate conversion efficiency.

The heights of the NCs are also the important parameters influencing the UCE of Si solar cells. As the top NCs and bottom NCs have different light-trapping performance, the optimized height of the top NCs and bottom NCs should be different from each other. For conformal NC structures, we change the height (H) of NCs from 200 to 500 nm gradually to find an optimal value of H for the thin-film solar cell. Here, the parameters are set as OT = 1.6 and a = b =300 nm. Figure 9a illustrates the relationship between UCE and H. The UCE reaches its maximum of 30.71% when H = 500 nm and the UCE also exceeds 30% when H = 300 nm, which derives from the tradeoff between antireflection and parasitic loss. Figure 9b shows the averaged reflection and averaged parasitic loss as a function of H. With the increase of H, the averaged reflection decreases due to GRI effect, while the parasitic loss increases. If H is too large, the incident light with longer wavelength will be absorbed by the Ag bottom NCs due to the LSPR effect. When H = 500 nm, the reduction of reflectance can compensate the parasitic loss, which leads to the highest UCE of 30.71%. With the increase of the H more than 500 nm, the UCE of conformal NCs will decrease again, because the parasitic loss will be enlarged greatly, which cannot be compensated by the corresponding enhanced light trapping.

**Figure 10:**
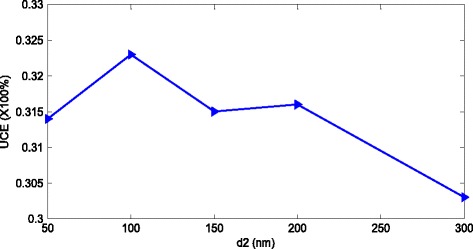
The UCE of the samples with d_1_ = 300 nm as the function of d_2_. UCE, ultimate conversion efficiency.

The height of the bottom NCs (d_2_) can be further optimized for the best light harvesting. We set d_1_ = 300 nm, OT = 1.6, a = b = 300 nm, and d_2_ changes from 50 to 300 nm gradually. The corresponding UCEs are shown in Figure 10. When d_2_ = 100 nm, the UCE reaches its maximum of 32.3% and the corresponding value is larger than that of optimal conformal NCs (d_1_ = d_2_ = 300 nm). The bottom NCs can trap the unabsorbed photons by SPRs and the scattering caused by the corrugation of the structure. However, if d_2_ is too large, there will be excessive parasitic loss which cannot be compensated by the enhanced light trapping. On the other hand, if d_2_ is too small, the bottom NCs cannot efficiently scatter or diffract the unabsorbed photons [23]. Therefore, there is a tradeoff between light trapping and parasitic loss.

**Figure 11:**
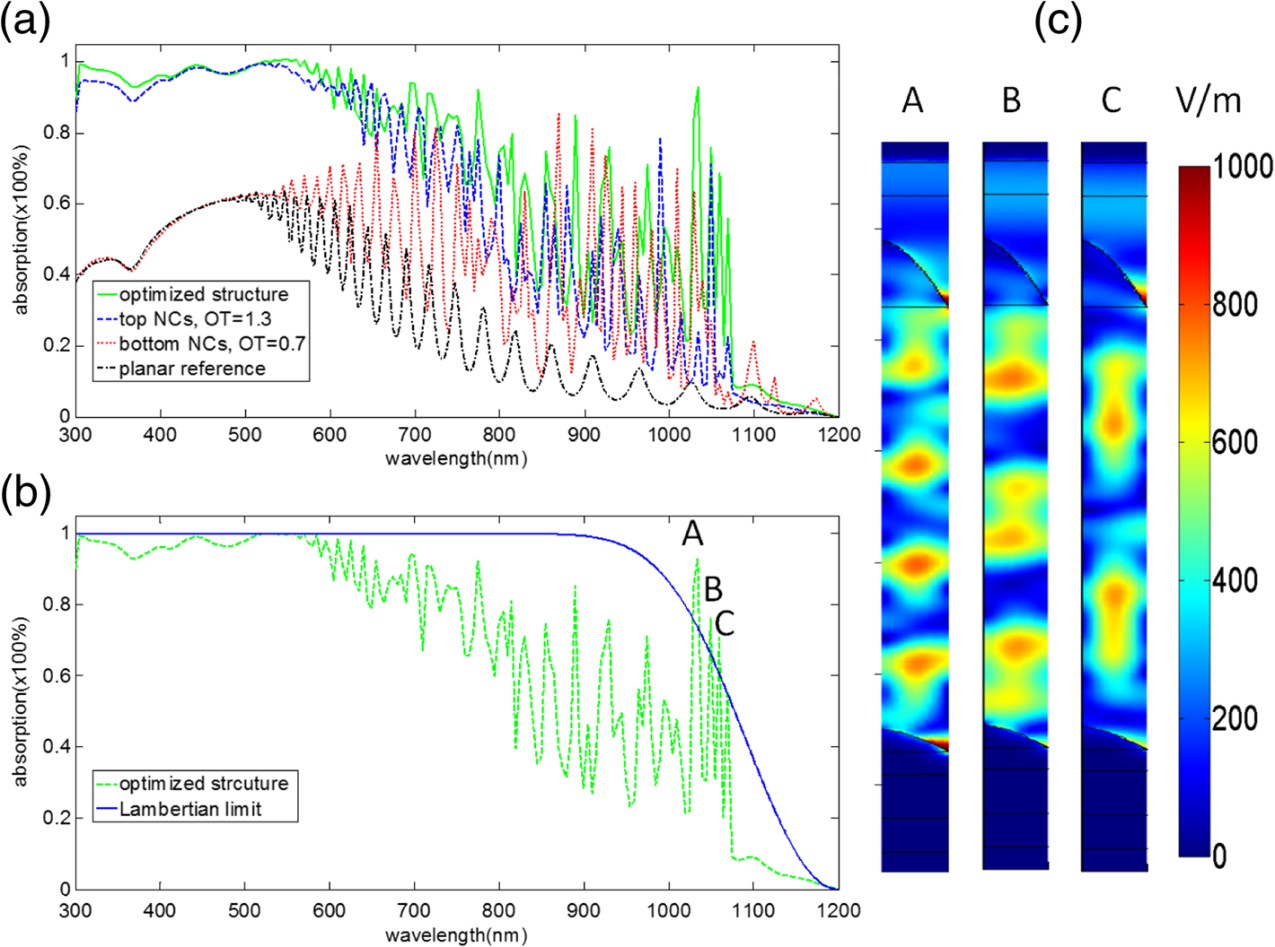
The absorption enhancement of different structures and the electric field intensity distribution at the absorption peaks. **(a)** The absorption spectra of solar cells with optimized dual NCs, optimal top NCs, and optimal bottom NCs with a reference of planar Si. **(b)** The absorption spectrum of the optimal dual NC solar cell and the Lambertian limit curve. **(c)** The electric field intensity distributions at the absorption peaks of A, B, and C in (b), respectively. NCs, nanocone arrays; OT, order of taper.

In order to characterize the absorption enhancement of the optimal dual photonic crystal clearly, we illustrate the absorption spectrum of optimal dual NCs, optimal bottom NCs, and optimal top NCs in Figure 11a with a reference of that of the planar Si thin-film solar cells (with the same depth as the formers). As shown in Figure 11a, the optimal bottom NCs can enhance the absorption in the longer wavelength region compared to the planar Si thin film, which can be ascribed to the multiple scattering effect and the excitation of LSPs of the bottom NCs. The top NCs can enhance the absorption in the whole wavelength region due to the GRI effect, multiple scattering diffraction, and waveguide modes coupling. For our optimal dual NCs, we can see that the absorption can be further enhanced in the whole wavelength region because of the combination of the mechanisms mentioned above.

As depicted in Figure 11b, the absorption spectrum of the optimal dual NCs has been also compared with Lambertian limit that can be obtained by calculating the following equation [31]:

8 $$ {\mathrm{A}}_{\mathrm{limit}}\left(\uplambda \right)=1-{\mathrm{e}}^{4n{\left(\uplambda \right)}^2\times \upalpha \left(\uplambda \right)\times \mathrm{d}} $$

where, *n*(λ) is the real part of the refractive index of Si at a specific wavelength, α(λ) is the absorption coefficient of Si and *d* is the thickness of solar cell.

From Figure 11b, the performance of the optimal dual NCs can achieve the Lambertian limit in the short wavelength region nearly. And it exceeds the Lambertian limit at the absorption peaks in 1,034, 1,049, and 1,059 nm (labeled as A, B, C, respectively) in the NIR region [32]. The result indicates that our optimized solar cell is efficient. In fact, some groups have also proved that using nanophotonics in solar cell can exceed the 4*n*^2^ limit [33,34] before. The electric field distributions corresponding to A, B, and C are illustrated in Figure 11c, respectively, which show the enhanced modes clearly. For these three peaks, the LSPs can be observed at the corner of the bottom NCs, and the incident light can be concentrated in the Si layer, which can enhance the light absorption.

## Conclusions

In conclusion, we have proposed a novel method for defining the shape of NC structures by a new equation. The shape of the proposed dual photonic crystal is investigated and optimized by the self-definition. For conformal NCs, the parabolic-shape Si nanostuctures with OT = 1.6 is the optimized choice, in which the corresponding UCE can reach to 30.3% with the thickness of only 2 μm. The shapes of top NCs and bottom NCs of the thin film have been investigated and discussed in detail by comparing with the planar Si thin-film solar cell respectively. The simulation result indicates that the top NCs play a more important role in light harvesting. The optimal OT for the top NCs is 1.3 and that of the bottom NCs is 0.7. The heights of top NCs and bottom NCs have been also optimized in detail. The optimal height of top NCs and bottom NCs differs from each other, and the optimal UCE is 32.3% when the period equals 300 nm.

## References

[1] Wangyang P, Wang Q, Wan X, Hu KX, Huang K (2013). Optical absorption enhancement in silicon square nanohole and hybrid square nanowire-hole arrays for photovoltaic application. Optics Commun.

[2] Huang H, Linfeng L, Wang J, Yang J, Leung S-F, Wang Y (2013). Performance enhancement of thin-film amorphous silicon solar cells with low cost nanodent plasmonic substrates. Energy Environ Sci.

[3] Biswas R, Chun X (2012). Photonic and plasmonic crystal based enhancement of solar cells–theory of overcoming the Lambertian limit. J Non Cryst Solids.

[4] Pratesi F, Burresi M, Riboli F, Vynck K, Wiersma DS (2013). Disordered photonic structures for light harvesting in solar cells. Opt Express.

[5] Burresi M, Pratesi F, Vynck K, Prasciolu M, Tormen M, Wiersma DS (2013). Two-dimensional disorder for broadband, omnidirectional and polarization-insensitive absorption. Opt Express.

[6] Xia Z, Yonggang W, Jiao H, Cao H, Liang Z, Zhou J (2014). Thin film silicon solar cells with non-simple integral period ratio between front and back gratings. J Opt.

[7] Nguyen-Huu N, Cada M, Pištora J (2014). Investigation of optical absorptance of one dimensionally periodic silicon gratings as solar absorbers for solar cells. Opt Express.

[8] Biswas R, Timmons E (2013). Nano-photonic light trapping near the Lambertian limit in organic solar cell architectures. Opt Express.

[9] Varghese LT, Xuan Y, Niu B, Fan L, Bermel P, Qi M (2013). Enhanced photon management of thin-film silicon solar cells using inverse opal photonic crystals with 3D photonic bandgaps. Adv Optical Mater.

[10] Pahud C, Isabella O, Naqavi A, Haug F-J, Zeman M, Herzig HP (2013). Plasmonic silicon solar cells: impact of material quality and geometry. Opt Express.

[11] Tan H, Santbergen R, Smets AHM, Zeman M (2012). Plasmonic light trapping in thin-film silicon solar cells with improved self-assembled silver nanoparticles. Nano Lett.

[12] Zhang X, Yu Y, Xi J, Liu T, Sun X-H (2015). The plasmonic enhancement in silicon nanocone hole solar cells with back located metal particles. J Opt.

[13] Jing Y, Shao W, Zhou Y, Wang H, Liu X, Xu X (2013). Nano Ag-enhanced energy conversion efficiency in standard commercial pc-Si solar cells and numerical simulations with finite difference time domain method. Appl Phys Lett.

[14] Lin A, Zhong Y-K, Ssu-Ming F (2013). The effect of mode excitations on the absorption enhancement for silicon thin film solar cells. J Appl Phys.

[15] Jung J-Y, Guo Z, Jee S-W, Um H-D, Park K-T, Kim C-T (2010). A waferscale Si wire solar cell using radial and bulk p–n junctions. Nanotechnology.

[16] Garnett E, Yang P (2010). Light trapping in silicon nanowire solar cells. Nano Lett.

[17] Jung J-Y, Guo Z, Jee S-W, Um H-D, Park K-T, Lee J-H (2010). A strong antireflective solar cell prepared by tapering silicon nanowires. Opt Express.

[18] Park K-T, Guo Z, Um H-D, Jung J-Y, Jee S-W, Yang JM (2011). Optical properties of Si microwires combined with small nanoneedles for flexible thin film photovoltaics. Opt Express.

[19] Zhu J, Hsu C-M, Zongfu Y, Fan S, Cui Y (2010). Nanodome solar cells with efficient light management and self-cleaning. Nano Lett.

[20] Kuo S-Y, Hsieh M-Y (2014). Efficiency enhancement in Cu_2_ZnSnS_4_ solar cells with subwavelength grating nanostructures. Nanoscale.

[21] Abass A, Le KQ, Alu A, Burgelman M, Maes B (2012). Dual-interface gratings for broadband absorption enhancement in thin-film solar cells. Physical Rev B.

[22] Peer A, Biswas R (2014). Nanophotonic organic solar cell architecture for advanced light trapping with dual photonic crystals. ACS Photonics.

[23] Jovanov V, Palanchoke U, Magnus P, Stiebig H, Hüpkes J, Sichanugrist P (2013). Light trapping in periodically textured amorphous silicon thin film solar cells using realistic interface morphologies. Opt Express.

[24] Palik ED (1985). Handbook of Optical Constants of Solids.

[25] Zhou K, Guo Z, Li X, Jung J-Y, Jee S-W, Park K-T (2012). The tradeoff between plasmonic enhancement and optical loss in silicon nanowire solar cells integrated in a metal back reflector. Opt Express.

[26] Zhang X, Sun X-H, Jiang L-D (2013). Absorption enhancement using nanoneedle array for solar cell. Appl Phys Lett.

[27] Zhou K, Li X, Liu S, Lee J-H (2014). Geometric dependence of antireflective nanocone arrays towards ultrathin crystalline silicon solar. Nanotechnology.

[28] Ferry VE, Polman A, Atwater HA (2011). Modeling light trapping in nanostructured solar cells. ACS Nano.

[29] Lin C, Povinelli ML (2009). Optical absorption enhancement in silicon nanowire arrays with a large lattice constant for photovoltaic applications. Opt Express.

[30] Zhang R, Shao B, Dong J, Zhang J, Yang H (2011). Absorption enhancement analysis of crystalline Si thin film solar cells based on broadband antireflection nanocone grating. J Appl Phys.

[31] Bozzola A, Liscidini M, Andreani LC (2012). Photonic light-trapping versus Lambertian limits in thin film silicon solar cells with 1D and 2D periodic patterns. Opt Express.

[32] Spinelli P, Verschuuren MA, Polman A (2012). Broadband omnidirectional antireflection coating based on subwavelength surface Mie resonators. Nat Commun.

[33] Zongfu Y, Raman A, Fan S (2010). Fundamental limit of nanophotonic light trapping in solar cells. Proc Natl Acad Sci U S A.

[34] Callahan DM, Munday JN, Atwater HA (2012). Solar cell light trapping beyond the ray optic limit. Nano Lett.

